# The Double-Edged Sword of Online Learning for Ethnoracial Differences in Adolescent Mental Health During Late Period of the COVID-19 Pandemic in the United States: National Survey

**DOI:** 10.2196/55759

**Published:** 2024-08-05

**Authors:** Celeste Campos-Castillo, Vijaya Tamla Rai, Linnea I Laestadius

**Affiliations:** 1 Department of Media and Information Michigan State University East Lansing, MI United States; 2 Department of Sociology and Anthropology Montana State University Bozeman, MT United States; 3 Zilber College of Public Health University of Wisconsin-Milwaukee Milwaukee, WI United States

**Keywords:** mental health, school modality, race and ethnicity, confidants, sleep, equity, remote learning, virtual learning, racial justice, anxiety, depression, depressive, student, students, school, schools, adolescent, adolescents, teen, teens, teenager, teenagers, race, racial, eLearning, online learning, education, equality, inequality, inequity, disparity, disparities, teaching, ethnic, ethnicities

## Abstract

**Background:**

Despite several theories suggesting online learning during the COVID-19 pandemic would aggravate ethnoracial disparities in mental health among adolescents, extant findings suggest no ethnoracial differences in mental health or that those from minoritized ethnoracial groups reported better mental health than their White counterparts.

**Objective:**

This study aimed to identify why findings from prior studies appear to not support that ethnoracial disparities in mental health were aggravated by testing 2 pathways. In pathway 1 pathway, online learning was associated with reporting fewer confidants, which in turn was associated with poorer mental health. In pathway 2, online learning was associated with reporting better sleep, which in turn was associated with better mental health.

**Methods:**

We analyzed survey data from a US sample (N=540) of 13- to 17-year-olds to estimate how school modality was associated with mental health via the 2 pathways. The sample was recruited from the AmeriSpeak Teen Panel during spring of 2021, with an oversample of Black and Latino respondents. Ethnoracial categories were Black, Latino, White, and other. Mental health was measured with the 4-item Patient Health Questionnaire, which assesses self-reported frequency of experiencing symptoms consistent with anxiety and depression. School modality was recorded as either fully online or with some in-person component (fully in-person or hybrid). We recorded self-reports of the number of confidants and quality of sleep. Covariates included additional demographics and access to high-speed internet. We estimated bivariate associations between ethnoracial group membership and both school modality and mental health. To test the pathways, we estimated a path model.

**Results:**

Black and Latino respondents were more likely to report being in fully online learning than their White counterparts (*P<*.001). Respondents in fully online learning reported fewer confidants than those with any in-person learning component (β=–.403; *P=*.001), and reporting fewer confidants was associated with an increased likelihood of reporting symptoms consistent with anxiety (β=–.121; *P=.*01) and depression (β=–.197; *P<*.001). Fully online learning respondents also reported fewer concerns of insufficient sleep than their in-person learning counterparts (β=–.162; *P*=.006), and reporting fewer concerns was associated with a decreased likelihood of reporting symptoms consistent with anxiety (β=.601; *P<*.001) and depression (β=.588; *P<*.001). Because of these countervailing pathways, the total effect of membership in a minoritized ethnoracial group on mental health was nonsignificant.

**Conclusions:**

The findings compel more nuanced discussions about the consequences of online learning and theorizing about the pandemic’s impact on minoritized ethnoracial groups. While online learning may be a detriment to social connections, it appears to benefit sleep. Interventions should foster social connections in online learning and improve sleep, such as implementing policies to enable later start times for classes. Future research should incorporate administrative data about school modality, rather than relying on self-reports.

## Introduction

### Overview

When US schools pivoted to online learning in March 2020 to curb the spread of SARS-CoV-2, concerns were raised about potentially harmful effects on children’s mental health [1,2]. Schools began returning to in-person learning in the fall of 2020, but adolescents and those from minoritized ethnoracial groups tended to remain in fully online learning [3-7], raising concerns about ethnoracial disparities in mental health. By “minoritized,” we refer to systemic oppression across a range of contexts by the majority group (in the United States, by White communities), and by “ethnoracial” we recognize that cultural groups are typically racialized, meaning treated as a race, and thus use a term that combines “ethnicity” and “race” [8]. Ethnoracial disparities are, therefore, inequalities along ethnic and racial group lines. Despite disproportionate exposure to online learning during the pandemic, several studies report that adolescents from minoritized ethnoracial groups either have comparable [9-12] or better [13-18] mental health than their White counterparts. Such paradoxical findings indicating that minoritized groups at times fare better contradict popular theories [19], including the fundamental cause theory [20]. What remains unknown is why such paradoxical findings exist.

This study aims to explain why such paradoxical findings exist. Resolving this gap in the literature is necessary to inform how best to design interventions to support student well-being and mitigate the potential for ethnoracial disparities. We investigate the possibility that it both constrained and enabled access to stress buffers, which are resources that limit stress from translating into poor mental health [21]. Specifically, we evaluate key stress buffers for adolescents [22,23] that are likely to be differentially associated with school modality, defined as the mode of instruction—confidants and sleep. We hypothesize that while being fully online is likely associated with access to fewer confidants relative to in-person learning, it may be associated with relatively better sleep. The persistent association between school modality and ethnoracial membership would thereby result in negligible ethnoracial differences in mental health, which provides 1 explanation for paradoxical findings on adolescent mental health during the pandemic.

We elaborate on the literature supporting these hypotheses and test our assertions using a national sample of US adolescents surveyed in the spring of 2021. We identify plausible explanations for why nonsignificant ethnoracial differences in mental health appear and ways to support the mental health of adolescents. Our findings suggest lessons for supporting student’s well-being and compel the need for more nuanced discussions and theorizing about the detriments and benefits of online learning for minoritized ethnoracial groups.

### Background

Despite concerns that online learning would harm children’s mental health, studies conducted in the United States during the early phase of the pandemic showed inconsistent results. For instance, a study of adolescents across the United States showed the shift created mental and emotional strain [10], while another study of adolescents in a majority Latino southwestern US school found significant reductions in mental health problems [24]. Another study analyzed a national sample of US adolescents recruited via social media and found no association between changes in mental health and school closures [25]. By the fall of 2020, when more schools began offering in-person learning, studies of ethnoracially diverse US samples began showing poorer mental health among students enrolled in online learning than in in-person learning [4-6,26]. Nonetheless, there were studies suggesting alternative relationships [27,28]. Such inconsistencies make it difficult to draw conclusions about how online learning may have impacted children’s mental health. Adding to the difficulties is that several studies suggesting a negative impact are unable to distinguish the impact of school modality from other lockdown measures [29].

The assertion that online learning harms mental health suggests that those most likely to be in online learning within the United States—adolescents from minoritized ethnoracial groups [3-6,26]—should report poorer mental health. Yet, studies find that either in-person learning harms [24,28] or school modality has no impact [27] on the mental health of those from minoritized ethnoracial groups. The evidence thus presents a paradox, wherein minoritized groups appear protected.

There are several possible methodological explanations for the paradox. For example, studies conducted early in the pandemic may have had insufficient time for stressors to accumulate and produce measurable differences. Differences in informants, that is, guardians (eg, [6,9,26]) versus adolescents themselves (eg, [5,27]), can also sway patterns because adolescents from minoritized ethnoracial groups may withhold mental health problems from guardians to avoid punishment or ridicule [30]. Several studies evaluating ethnoracial differences in mental health used new scales to capture pandemic-specific well-being concerns that may be susceptible to ethnoracial bias [31]. Many studies also lacked nationally representative samples (eg, [24,27]) and may represent the impact of only local pandemic policies.

This study is a step toward explaining the paradox by making several key methodological maneuvers. We surveyed a nationally representative sample of adolescents during a later period of the pandemic using a mental health scale that is validated for use among ethnoracially diverse adolescents and ethnoracial invariance, meaning it appears to be not susceptible to ethnoracial bias [32-34]. We also evaluated mechanisms by which school modality may contribute to ethnoracial differences in mental health, thereby addressing a gap in extant research [17,18,35]. Next, we outline reasons for hypothesizing that during later periods of the pandemic, online learning could both harm and benefit adolescent mental health via modulating access to stress buffers. A deeper consideration that online learning may be a “double-edged sword” can help resolve this mental health paradox, while also pointing to the policies and interventions that may help support well-being.

### Hypotheses

**Figure 1:**
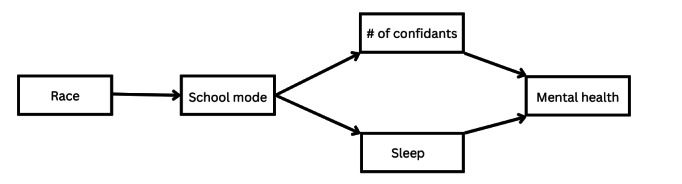
Summary of pathways linking modality to mental health among adolescents.

Figure 1 summarizes the mechanisms we test that link ethnoracial group membership to mental health via school modality. Each describes a stress buffer (confidants and sleep) that is differentially associated with school modality and becomes key for protecting mental health in adolescence [22,23]. Pathway 1 (harm) hypothesizes fully online learning can harm adolescent mental health because it is associated with adolescents reporting fewer confidants. Pathway 2 (benefit) hypothesizes fully online learning is beneficial for adolescent mental health because it is associated with adolescents reporting better sleep. Because adolescents from minoritized ethnoracial groups are more likely to be in online learning than their White counterparts, pathway 1 (harm) would yield estimates where they report relatively worse mental health, while this pattern would be reversed for pathway 2 (benefit). Importantly, an analysis that estimates both paths simultaneously and finds support that both are operating can result in an overall nonsignificant association between ethnoracial group membership and mental health. This would help explain the paradox of nonsignificant ethnoracial differences in adolescent mental health during the pandemic. We also estimated a direct path from school modality to mental health (not shown in the figure) to capture any remaining association.

The first hypothesis reflects the persistent ethnoracial variation in school modality during the pandemic. When schools began reopening during the 2020-2021 academic year, adolescents from minoritized ethnoracial groups were less likely than their White counterparts to experience in-person learning, including hybrid forms that combined online with in-person learning [3-6,26]. This was partly attributable to the choices of Black and Latino families, who were disproportionately impacted by the COVID-19 pandemic and thereby concerned about their children getting infected during in-person learning [3,36,37], but also to geographic segregation. Starting from the fall of 2020, the presence of any in-person learning was unrelated to local COVID-19 case counts and instead became more common in nonmetropolitan areas, Republican-leaning school districts, and school districts with weaker teachers’ unions [3,38-42]. Due to segregation, Black and Latino families are less likely than their White counterparts to live in these areas, resulting in a stronger tendency to be in fully online learning [3,39,42].

Adolescents from minoritized racial and ethnic groups are significantly more likely than their White counterparts to be in fully online learning.

H1

As Figure 1 shows, we evaluate 2 countervailing mechanisms linking school modality to mental health, whereby 1 pathway describes how being fully online constrains access to a stress buffer while another describes how it facilitates access to a stress buffer.

### Pathway 1: How Online Learning Can Harm Adolescent Mental Health

In the first pathway, fully online learning can harm adolescent mental health by limiting access to social ties, specifically confidants. Schools exert influence on youth by providing a context in which social relationships are nurtured and confer resources [43]. At schools, youth can cultivate ties to confidants, including peers, teachers, other school staffs, and parents of peers [30,44]. Notably, opportunities for tie formation are likely more common in in-person settings. For example, organizational routines, dictating pickup and drop-off times and locations, and structure opportunities for people to be physically collocated and thereby form ties spontaneously [45].

Social connections are still possible through technology-mediated interactions [46-48]. Particularly during the pandemic, adolescents rely on technologies (eg, social media, video chat, and text messaging) to connect with others [13,25,49]. However, these connections are largely buttressed by ties made via physical school settings [50-52]. Therefore, we suggest that restricting access to a key physical location where adolescents socialize—the school—makes it more difficult to cultivate ties. Notably, our measure for confidants did not restrict respondents to only reporting others with whom they engage in person.

Adolescents in fully online learning will report fewer confidants than those with any in-person learning component (fully in-person, hybrid).

H2

The support confidants provide, including those cultivated via schools, can help buffer against stress [30,53-55]. Support includes social support to cope with personal problems and instrumental support that assists with accessing mental health care. As children progress through adolescence, they increasingly rely on their school peers as confidants [23]. Indeed, research during the pandemic shows access to social relationships such as confidants can mitigate mental health concerns among adolescents [5,10,13].

Adolescents will report better mental health when they report a greater number of confidants.

H3

### Pathway 2: How Online Learning May Benefit Adolescent Mental Health

The second pathway evaluates the potential benefits of fully online learning for adolescent mental health via promoting better sleep. Research during the pandemic shows online learning is associated with longer sleep duration than in-person learning, even when start times are the same because sleep gains can arise from no longer needing to account for travel [56]. Students in online learning may also sleep better from less rumination over bullying in school, since online learning reduced adolescents’ exposure to bullying [57-59]. Moreover, hybrid modes disrupted sleep relative to being fully online because students were frustrated with having to remember when they had to be in person versus online [60].

Adolescents in fully online learning will report better sleep than those with an in-person learning component.

H4

Sleep quality is positively associated with a range of adolescent health outcomes, including better mental health. Previously, researchers assumed the association was solely because poor mental health caused sleep disruption, but longitudinal studies and randomized control trials show causality is most likely occurring in the reverse direction [61,62]. Sleep operates as a stress buffer and this appears particularly important as children enter adolescence [22]. Adequate sleep promotes positive affect and emotional regulation to process negative affect and limit rumination and worry, which over time can reduce the risk of developing anxiety and depression [62].

Adolescents who report better sleep will report better mental health.

H5

## Methods

### Pretesting

Prior to fielding the 10-minute English survey that covered mental health, technology use, and pandemic experiences, we conducted cognitive interviews via Zoom (Zoom Video Communications) with 10 adolescents aged 13 to 17 years from across the United States during December 2020 and January 2021. While key measures such as mental health are drawn from validated instruments, the cognitive interviews provided feedback on whether survey wording aligned with pandemic experiences (options for school modality and confidant connections) and content validity of single-item measures (confidants and sleep). We opted for single-item measures, where possible, to reduce respondent burden. While multiple-item scales are generally preferred over single items to measure a construct, single items can perform just as well for well-understood topics [63], which the cognitive interviews enabled checking. The survey text was revised as needed. Specific to this study, we revised the school modality question to specify we were only interested in the modality for high school courses since some interviewees indicated they were also enrolled in college courses. Each interview lasted approximately 1 hour, and interviewees received US $40 gift cards.

### Survey Respondents

From March to May 2021, the National Opinion Research Center (NORC) at the University of Chicago invited 784 adolescents aged 13 to 17 years from their AmeriSpeak Teen Panel to complete the online survey, with 540 (68.9% completion rate) responding. AmeriSpeak is a probability-based panel designed to be representative of US households. For the purposes of this survey, NORC oversampled Black and Latino respondents. This limits our ability to generalize to the full population of adolescents but strengthens our ability to compare patterns by ethnoracial groups. We analyzed the path model with a full information estimator, which enables using all information available from the 540 respondents. The measure with the most missing values was school modality, which had 19 (3.5%) missing values. Sample characteristics are described in the Results section.

### Ethical Considerations

This research was approved by the institutional review board at NORC (approval number FWA00000142). NORC collected consent from parents or guardians and assent from adolescents prior to administering the survey. Participants were compensated US $5 for their time. Deidentified data were shared with the authors.

### Measures

#### Race and Ethnicity

NORC provided the race and ethnicity of adolescents, which they collected when constructing the AmeriSpeak panel. We use 4 mutually exclusive categories—Black, Latino, White, and other. The first 3 were predefined by NORC. We constructed the other category to combine those who identified as Asian Americans, Indigenous people, and multiple races because of their small sample sizes.

#### School Modality

Respondents indicated their current mode of high school classes. We compared those who were fully online (coded as 1) to those who had an in-person learning component (coded as 0), which included being fully in person and in a hybrid or combined format.

#### Mental Health

Respondents reported their mental health using the 4-item Patient Health Questionnaire (PHQ-4), which is validated for use among adolescents as a screener and has shown measurement invariance across ethnoracial groups [32-34]. The scale asks the frequency of experiencing mental distress (0=not at all to 3=nearly every day) during the 2 weeks prior to the survey, with 2 items referring to anxiety symptoms (α=.90) and 2 to depressive symptoms (α=.82). Items are summed to create 2 indexes, each ranging between 0 and 6 with higher numbers indicating higher levels of mental distress. Each score is then dichotomized, with respondents scoring 3 or greater coded as likely experiencing the condition represented by the subscale [34].

#### Sleep

We developed a single item resembling those appearing in questionnaires designed to ascertain sleep quality in samples that are ethnoracially diverse and include adolescents [64-66]. It asked the frequency with which respondents were concerned about “not getting enough sleep” over the past 2 weeks on a 4-point scale (0=not at all to 3=nearly every day), where higher values indicated poorer quality sleep.

#### Confidants

A single item asked respondents the number of people in the past month they could

talk to about things that are important to [them]—someone [they could] count on for understanding or support.

The item has been administered in other surveys of ethnoracially diverse individuals to understand how social support is associated with health outcomes [67].

#### Covariates

Based on the findings of previous studies [26,68,69], we adjusted for respondents’ age (in years), sex (female vs male), access to high-speed internet (yes vs no), household located within a metropolitan area (yes vs no), household size, and annual household income (logged).

#### Analysis

After describing the sample, we conducted bivariate analyses (chi-square tests) to evaluate whether there was significant ethnoracial variation in reports of school modality and mental health. To test hypotheses, we conducted a path analysis, which estimates regressions for the different hypothesized outcomes (school modality, sleep, confidants, anxiety, and depression) simultaneously. Each path in our heuristic model depicted in Figure 1 represents a regression estimated. Because regressions are estimated simultaneously, this is an improvement from single regression equations in 2 ways. First, it enables calculating effects between variables that are indirectly connected (eg, the effect of respondents’ race and ethnicity on their anxiety and depression). Second, it enables decomposing the sources by tracing the paths connecting them (eg, the 2 pathways explaining the effect of school modality—sleep and confidants) and evaluating their statistical significance.

Sample descriptives and bivariate analyses were computed using Stata (version 17.0; StataCorp). The path analysis was conducted in MPlus (version 8.6; Muthen and Muthen) using the weighted least squares means and variance adjusted estimator, which is appropriate when outcomes are not normally distributed, as in the case of categorical outcomes. Missing data were handled using full information maximum likelihood, which uses all information available to derive estimates. Full information maximum likelihood produces more efficient estimates (ie, better represents the true values) than other methods of handling missing values, such as listwise deletion, pairwise deletion, and multiple imputations [70]. Model fit was determined by evaluating the chi-square statistic, the comparative fit index (CFI), the Tucker-Lewis Index (TLI), and the root-mean-square error of approximation (RMEA). Significance tests were 2-sided with α=.05.

## Results

### Sample Characteristics

Table 1 shows a summary of sample characteristics. The ethnoracial breakdown was 15.4% (n=83) Black, 21.7% (n=117) Latino, 51.7% (n=279) White, and 11.3% (n=61) other races. A total of 43.3% (n=234) of respondents reported being in fully online classes, 32.2% (n=174) reported symptoms suggestive of anxiety, and 28.9% (n=156) reported symptoms suggestive of depression.

**Table 1:** Descriptives for sample of US adolescents surveyed in the spring of 2021.

Measure			Values
**Ethnoracial identity, n (%)**			
	Black	83 (15.4)	
	Latino	117 (21.7)	
	Other	61 (11.3)	
	White	279 (51.7)	
Fully online learning, n (%)			234 (44.9)
Number of confidants, mean (SD)			3.3 (2.3)
**Sleep quality, n (%)**			
	Not at all	203 (38.4)	
	Several days	163 (30.8)	
	More than half the days	88 (16.6)	
	Nearly every day	75 (14.2)	
Has symptoms consistent with anxiety, n (%)			174 (32.2)
Has symptoms consistent with depression, n (%)			156 (28.9)
Age, mean (SD)			15.3 (1.3)
Female, n (%)			297 (55.0)
Annual income (in thousands), mean (SD)			52.3 (25.4)
Household size, mean (SD)			4.9 (1.1)
Lives in metropolitan area, n (%)			447 (82.8)
Has broadband internet, n (%)			487 (90.2)

### Bivariate Analysis

Table 2 shows results from initial tests to determine whether respondents’ school modality and mental health varied significantly with their race and ethnicity. Respondents’ reports of being fully online varied significantly by ethnoracial membership (*χ*^2^_3_=22.1; *P*<.001). Specifically, being fully online was most common among Black (n=49, 62%) and Latino (n=61, 55%) respondents. Stated differently, compared to White respondents, Black respondents had 34% lower odds and Latino respondents had 47% lower odds of being in person. Neither reports of anxiety nor depression varied significantly by respondents’ ethnoracial membership. Not shown is that reports of being fully online were not significantly associated with reports of anxiety and depression.

**Table 2:** Bivariate analysis examining differences in school modality and mental health by ethnoracial identity of US adolescents in the spring of 2021.

	Ethnoracial identity					*P* value^a^
	Black, n (%)	Latino, n (%)	Other, n (%)	White, n (%)		
Fully online	49 (62.0)	61 (54.5)	26 (44.8)	98 (36.0)	<.001	
Anxiety	25 (30.1)	39 (33.3)	18 (29.5)	92 (33.0)	.92	
Depression	21 (25.3)	42 (35.9)	15 (24.6)	78 (28.0)	.26	

^a^*P* value is for chi-square test.

### Path Model

The model fits the data adequately [71]. While the chi-square statistic was significant (*χ*^2^_36_=67.516; *P<*.01), the CFI was above 0.90 (0.955), the TLI was above 0.90 (0.931), and the RMEA was below 0.050 (0.040). Standardized estimates for the paths appear in Table 3.

**Table 3:** Standardized coefficients for path model analyzing responses of US adolescents in the spring of 2021^a^.

			Dependent variable																		
Independent variable			Fully online learning, β (SE)		*P* value^b^		Number of confidants, β (SE)		*P* value^b^		Sleep, β (SE)		*P* value^b^		Anxiety, β (SE)		*P* value^b^		Depression, β (SE)		*P* value^b^
**Ethnoracial identity (vs White)**																					
	Black	.622 (0.170)		<.001		N/A^c^		N/A		N/A		N/A		N/A		N/A		N/A		N/A	
	Latino	.438 (0.151)		.004		N/A		N/A		N/A		N/A		N/A		N/A		N/A		N/A	
	Other	.206 (0.176)		.242		N/A		N/A		N/A		N/A		N/A		N/A		N/A		N/A	
Fully online learning			N/A		N/A		–.403 (0.119)		.001		–.162 (0.058)		.006		–.079 (0.068)		.248		.111 (0.068)		.109
Number of confidants			N/A		N/A		N/A		N/A		N/A		N/A		–.121 (0.049)		.014		–.197 (0.049)		<.001
Sleep quality			N/A		N/A		N/A		N/A		N/A		N/A		.601 (0.046)		<.001		.588 (0.044)		<.001
Age			.054 (0.045)		.234		N/A		N/A		N/A		N/A		N/A		N/A		N/A		N/A
Female			–.098 (0.114)		.389		N/A		N/A		N/A		N/A		N/A		N/A		N/A		N/A
Annual household income (logged)			–.103 (0.098)		.291		N/A		N/A		N/A		N/A		N/A		N/A		N/A		N/A
Household size			–.018 (0.049)		.717		N/A		N/A		N/A		N/A		N/A		N/A		N/A		N/A
Lives in metropolitan area			.220 (0.154)		.154		N/A		N/A		N/A		N/A		N/A		N/A		N/A		N/A
Has broadband internet			.011 (0.192)		.954		N/A		N/A		N/A		N/A		N/A		N/A		N/A		N/A

^a^Model fit: *χ*^2^_36_=67.5 *P<*.01; comparative fit index=0.955; Tucker-Lewis Index=0.931; root-mean-square error of approximation=0.04.

^b^*P* values of 2-tailed tests.

^c^N/A: not applicable.

The first test evaluated ethnoracial variation in school modality, adjusting for covariates. We find support for H1, whereby Black (β=.622; *P<*.001) and Latino (β=.438; *P=*.004) adolescents were significantly more likely than their White counterparts to report being in fully online learning. Respondents we grouped into the other race category were just as likely as White respondents to be in fully online learning.

We next examined how ethnoracial variation in school modality may channel mental health patterns. To evaluate the presence of additional mechanisms not tested explicitly, the path analysis included an estimate of the direct effect of school modality on both mental health outcomes, which Table 3 shows was nonsignificant. Any association between school modality with mental health in this sample, then, is likely to operate via the pathways we outlined.

### Evaluation of Pathway 1 (Mental Health Harms From Fully Online Learning)

Pathway 1 describes potential harm, via reporting fewer confidants, from being fully online. Respondents reported fewer confidants when they said they were in fully online learning (β=–.403; *P=*.001), supporting H2. This has implications for mental health because reporting a greater number of confidants is associated with a decreased likelihood of reporting symptoms consistent with both anxiety (β=–.121; *P*=.01) and depression (β=–.197; *P<*.001), which support H3.

### Evaluation of Pathway 2 (Mental Health Benefits From Fully Online Learning)

Pathway 2 describes the potential benefits of being fully online via better sleep. Respondents who said they were fully online reported significantly fewer days of being concerned that they were getting insufficient sleep than their in-person counterparts (β=–.162; *P=*.006), supporting H5. This has implications for mental health because those who reported more days with these concerns were more likely to report symptoms consistent with both anxiety (β=.601; *P<*.001) and depression (β=.588; *P<*.001), supporting H5.

### Decomposing Association Between Race and Ethnicity and Mental Health

The decomposition analysis summarized in Table 4 traced the 2 hypothesized pathways linking respondents’ ethnoracial membership to their mental health and tested the statistical significance. All pathways operated via school modality, thereby evaluating the mental health impacts that stemmed from the increased likelihood of adolescents from minoritized ethnoracial groups over their White counterparts to state they were fully online. Since respondents who were categorized in the other race category were neither no more nor less likely to be in online learning compared to White respondents, we only evaluated the pathways for Black and Latino respondents. All estimates are standardized.

**Table 4:** Decomposition of total effect of ethnoracial identity on mental health into the 2 indirect pathways^a^.

		Black, β (SE)	Latino, β (SE)
**Total effect of ethnoracial identity**			
	Anxiety	–.087 (0.048)	–.061 (0.036)
	Depression	.036 (0.044)	.025 (0.031)
**Pathway 1: harms of being fully online via fewer confidants**			
	Anxiety	.013 (0.007)	.009 (0.006)
	Depression	.022 (0.010)^b^	.015 (0.008)
**Pathway 2: benefits of being fully online via better sleep**			
	Anxiety	–.053 (0.025)^b^	–.037 (0.020)
	Depression	–.052 (0.026)^b^	–.037 (0.020)

^a^Total effects include path estimating the direct effect of being fully online on the mental health outcomes. When this path is removed from the model, the total effects remain nonsignificant.

^b^*P<*.05, 2-tailed test.

Consistent with a paradox, Table 4 shows the total effect of respondents’ race and ethnicity on their mental health reports was nonsignificant. Estimates for the total effect include the path estimating the direct effect of being fully online on mental health reports, which we reported earlier as nonsignificant. Not shown is that when this path is removed from the model, the total effect remains nonsignificant.

The rest of Table 4 shows evidence that the nonsignificant associations between respondents’ race and ethnicity and their mental health reports are due to countervailing pathways. Among Black respondents, there were significant indirect effects of their racial group membership on their reports of symptoms consistent with both anxiety and depression. For depression, pathway 1 was statistically significant (*P<*.05), whereby being fully online was associated with reporting fewer confidants and, in turn, an increased likelihood of reporting symptoms. Pathway 2 was also statistically significant (*P<*.05), indicating a decreased likelihood of reporting symptoms because their tendency to report being fully online was associated with fewer concerns about getting enough sleep. For anxiety, pathway 1 was only marginally significant (*P<*.10) while Pathway 2 was statistically significant (*P<*.05). Among Latino respondents, the patterns for both pathways paralleled those for Black respondents, but were only marginally significant (*P<*.10). Although only marginally significant, the fact that the pathways operated in opposing directions among Latino respondents is still notable for interpreting patterns in this study and others, which we elaborate on in the Discussion section.

### Supplemental Analyses

These substantive conclusions were held when we tested 3 alternative models, which we summarize here with estimates in Multimedia Appendix 1. In the first alternative model (Table S1 in Multimedia Appendix 1), rather than imposing cutoffs on the mental health outcomes and treating them as categorical variables, we treated them as continuous variables and estimated the indexes (ranging from 0 to 6) as the outcomes.

In the second alternative model (Table S2 in Multimedia Appendix 1), we evaluated whether the patterns observed here were spurious, given prior research documenting racial and ethnic disparities in adolescents’ confidant patterns and sleep quality [23,72]. By this logic, the significant relationships between school modality and both confidants and sleep quality may be attributable to all 3 sharing significant associations with race and ethnicity. Accordingly, this was a just-identified model (df=0) in which each path was adjusted for respondents’ race and ethnicity along with the other covariates.

In the third alternative model (Table S3 in Multimedia Appendix 1), we evaluated whether state-level context may explain racial and ethnic patterns. Based on respondents’ states and survey dates, we merged into the data indicators of state-level COVID-19 risk (the cumulative number of state-level COVID-19 cases, deaths, and vaccinations; whether a state-wide mask mandate was in place; whether school districts decided on their own to reopen) and political context (political leaning of state legislature and governor, percentage of presidential votes for Trump in 2020). Data for the latter came from the Massachusetts Institute of Technology Election Data Science Lab [73] and the National Conference of State Legislatures [74], while the former from the Centers for Disease Control and Prevention [75] and Education Week [76]. Consistent with other studies conducted around the spring of 2021 [3,38,40], these variables did little to explain ethnoracial disparities in school modality.

Altogether, findings from these alternative models suggest the model summarized in the main text is a parsimonious approximation of the relationships in these data.

## Discussion

### Principal Findings

To understand how high rates of online learning among US adolescents from minoritized ethnoracial groups during the pandemic impacted mental health and ethnoracial disparities, we conducted a path analysis using data from a national sample. Results suggest online learning both constrained and facilitated access to stress buffers—confidants and sleep—revealing 2 countervailing pathways that link ethnoracial group membership to mental health. Adolescents in fully online learning reported fewer confidants, which was associated with poorer mental health. They also reported better quality sleep, which was associated with better mental health. Because adolescents from minoritized ethnoracial groups were most likely to be in fully online learning, these paths offset each other and produced negligible ethnoracial differences in mental health. The patterns held when we examined alternative explanations, including the role of political context. The findings suggest important implications for making sense of paradoxical findings in studies of pandemic mental health among adolescents and the potential impact of online learning, while also highlighting lessons for supporting student mental health.

Our findings highlight the importance of disaggregating multiple mechanisms that underlie outcomes to avoid drawing false conclusions. For example, in the bivariate analysis and estimates of total effects in the path analysis, there were no significant differences in mental health by school modality. This conflicts with previous research showing online learning has a negative association with mental health (eg, [10,26]). Several methodological differences exist between this study and previous ones that can plausibly explain the conflicting patterns. For example, previous studies collected data earlier in the pandemic when online learning was perhaps more disruptive to all, regardless of race and ethnicity. However, the net negative association in these other studies could have resulted from the covariates in the model, as scholars have noted that buffering effects can get obfuscated [21], or from confounding lockdown measures contributing negative effects [29]. Analyzing data using a path model and during a time when lockdown measures were being eased, provides a clearer assessment than in previous research about the possibility that online learning has both a negative and positive influence on mental health. Indeed, the possibility that some mental health benefits existed in these previous studies is bolstered by additional studies conducted around the same time showing online learning was associated with better mental health [24] and that mental health declined when students returned to in-person learning (eg, [77,78]). Future data collection efforts should consider potential benefits from online learning and revisit analyses from past efforts using models such as ours to directly assess both benefits and detriments.

We also demonstrated how without disaggregating mechanisms, results can suggest a paradox, whereby a group facing compounding disadvantages during the pandemic—adolescents from minoritized ethnoracial groups—appeared to report mental health comparable to their more advantaged counterparts. While this accords with several studies reviewed earlier, it constitutes a paradox because it conflicts with the propositions of several theories (eg, [20]). Disaggregating mechanisms, as we did, is critical for adjudication. Adolescents from minoritized ethnoracial groups do face compounding disadvantages, as suggested in the findings showing they are more likely than White adolescents to be in fully online learning, which is associated with reporting fewer confidants and poorer mental health. However, they may also experience advantages, such as better sleep and mental health, which were associated with being fully online. The suggested advantages of being fully online are held even with alternative specifications of the path model. Families from minoritized ethnoracial groups may prefer fully online learning partially to buffer against stressors and health risks stemming from compounding disadvantages, such as greater risks of severe COVID-19 complications and school discrimination [36,37,79,80]. While we cannot test this assertion here, we encourage a search for evidence that disadvantaged groups purposely seek out buffers such as online learning to offset risks.

Findings hold important implications for designing interventions and policies to support students based on school modality, particularly since schools will continue to prepare for crises necessitating emergency shifts to online learning and some families prefer to stay online rather than return to in-person learning [81]. Like others, we show patterns suggesting that access to technologies during the pandemic may positively benefit adolescents from minoritized ethnoracial groups [82]. To harness potential benefits, policy makers need to ensure access to devices and reliable, high-speed internet, such as funding programs that loan devices and support the construction of broadband infrastructure. In addition to meeting learning objectives online, schools should also consider adolescents’ social needs to support mental health [4,35]. While much attention has focused on learning loss during the pandemic, particularly in the context of ethnoracial disparities [83], the social implications of online learning should not be neglected. Social connectedness online is possible [46-48], although structured online class time alone may be insufficient for providing social and emotional support [4,84]. Interventions may include teachers making explicit statements signaling the availability of emotional support and opportunities to break out of task-focused activities and engage in tangential banter, which students missed during online learning [85]. Such efforts may be particularly important for adolescents from minoritized ethnoracial groups, who are less likely than their White counterparts to feel as if their teachers are a source of social support [30]. Further research is needed to determine how schools can best intervene in cultivating confidants during the times when online learning is either necessary or preferred.

Our findings indicate that students in in-person learning also require mental health interventions but of a different kind. Respondents in our sample who were in fully online learning may have reported better sleep than those in in-person learning for several reasons. For example, they may have been able to sleep longer with a later start time or because they did not have to account for traveling to school. Despite the American Academy of Sleep Medicine calling for later start times to support student mental health, among other positive outcomes [86], barriers such as school bus system logistics have prevented changes to start times [87]. Other potential reasons for better sleep include a reduction in experiencing victimization from peers in the physical school setting [57-59]. While we cannot determine why being fully online is associated with better sleep, it is likely a complex set of processes that are entwined with racialized experiences. For example, adolescents from minoritized ethnoracial groups are more likely to experience discrimination in schools than their White counterparts [80,88], and their families face heightened COVID-19–related risks [7]. Consequently, their sleep may benefit more from being fully online. In addition to educational policies to support later start times, it is crucial for health care providers to encourage better sleep among adolescents.

### Limitations

Findings should be interpreted alongside study limitations. Survey data come from a cross-sectional design and, therefore, cannot indicate causal direction. For example, mental health concerns may cause some to select fully online learning options. While 1 study of Canadian children using an experimental design suggests this tendency does not fully explain the relationship between mental health and the school modality [89], additional research is needed to understand the US context. The self-reported data can introduce measurement bias, which we tried to mitigate by conducting cognitive interviews, but future research should examine complimentary data sources, such as tracking sleep patterns with wearable devices. Finally, the survey did not oversample Asian adolescents. Based on reports showing they faced heightened discrimination in schools when they began reopening [88], it is likely that our estimates regarding the benefits of being fully online are conservative.

### Conclusions

Despite these limitations, this study demonstrates the benefits of examining multiple mechanisms simultaneously to unravel paradoxes. The findings add nuance to debates about which modality is best for addressing health disparities and underscore the need for collaborative community efforts to ensure equity [7]. Online learning can harm the mental health of adolescents, but it can also support it, so long as resources are mobilized accordingly. Discovering the mechanisms linking school modality to mental health enables pinpointing where to deploy resources in the event of a situation necessitating online learning. Extended public health measures are needed to support educational policies such as later school start times, addressing discrimination and victimization concerns in schools, and providing opportunities to cultivate social ties in online learning environments. Collaborative community development will likely be necessary to act on the insights gleaned from analytic approaches that uncover multiple operating mechanisms that can either facilitate or constrain access to stress buffers.

## Appendix

Multimedia Appendix 1 Results from supplemental analyses.

## Abbreviations

CFI: comparative fit index
NORC: National Opinion Research Center
PHQ-4: 4-item Patient Health Questionnaire
RMEA: root-mean-square error of approximation
TLI: Tucker-Lewis Index

## Data Availability

The data sets generated and analyzed during this study are not publicly available due to participants not consenting to their public release but are available from the corresponding author on reasonable request.
