# Supplementary material for: The Double-Edged Sword of Online Learning for Ethnoracial Differences in Adolescent Mental Health During Late Period of the COVID-19 Pandemic in the United States: National Survey
Source: JMIR Form Res. 2024 Aug 5;8:e55759. doi: 10.2196/55759 (PMC11333869; doi:10.2196/55759)
Supplement: Multimedia Appendix 1 [file formative_v8i1e55759_app1.docx]

| **Table S1.** Standardized estimates for path model with anxiety and depression measured as continuous variables. | | | | | | | | | | |
| --- | --- | --- | --- | --- | --- | --- | --- | --- | --- | --- |
|  |  |  | Dependent Variable | | | | | | | |
| Independent variable | Fully online learning |  | Number of confidants |  | Sleep |  | Anxiety |  | Depression | |
| Ethnoracial identity (vs. White) |  |  |  |  |  |  |  |  |  |  |
| Black | .570 | *** |  |  |  |  |  |  |  |  |
|  | (.161) |  |  |  |  |  |  |  |  |  |
| Latino | .375 | ** |  |  |  |  |  |  |  |  |
|  | (.144) |  |  |  |  |  |  |  |  |  |
| Other | .179 |  |  |  |  |  |  |  |  |  |
|  | (.168) |  |  |  |  |  |  |  |  |  |
| Fully online learning |  |  | -0.183 | ** | -.154 | ** | -.052 |  | .062 |  |
|  |  |  | (.054) |  | (.058) |  | (.049) |  | (.046) |  |
| Number of confidants |  |  |  |  |  |  | -.089 | ** | -.133 | *** |
|  |  |  |  |  |  |  | (.034) |  | (.037) |  |
| Sleep quality |  |  |  |  |  |  | .543 | *** | .570 | *** |
|  |  |  |  |  |  |  | (.035) |  | (.035) |  |
| Age | .059 |  |  |  |  |  |  |  |  |  |
|  | (.055) |  |  |  |  |  |  |  |  |  |
| Female | -.114 |  |  |  |  |  |  |  |  |  |
|  | (.110) |  |  |  |  |  |  |  |  |  |
| Annual household income (logged) | -.064 |  |  |  |  |  |  |  |  |  |
|  | (.059) |  |  |  |  |  |  |  |  |  |
| Household size | -.024 |  |  |  |  |  |  |  |  |  |
|  | (.054) |  |  |  |  |  |  |  |  |  |
| Lives in metropolitan area | .248 |  |  |  |  |  |  |  |  |  |
|  | (.148) |  |  |  |  |  |  |  |  |  |
| Has broadband internet | .001 |  |  |  |  |  |  |  |  |  |
|  | (.186) |  |  |  |  |  |  |  |  |  |
| Note: Numbers in parentheses are standard errors. Model fit: χ2 = 69.225, df = 36, p < .001; CFI = 0.894; TLI = 0.838; RMSEA = 0.041. | | | | | | | | | | |
| *p < .05, **p < .01, and ***p < .001 (two-tailed tests). | | | | | | | | | | |

| **Table S2.** Standardized estimates for path model with demographic variables entered as covariates for each path. | | | | | | | | | | |
| --- | --- | --- | --- | --- | --- | --- | --- | --- | --- | --- |
|  | Dependent Variable | | | | | | | | | |
| Independent variable | Fully online learning |  | Number of confidants |  | Sleep |  | Anxiety |  | Depression |  |
| Ethnoracial identity (vs. White) |  |  |  |  |  |  |  |  |  |  |
| Black | .578 | *** | .040 |  | -.041 |  | .021 |  | -.016 |  |
|  | (.159) |  | (.134) |  | (.140) |  | (.155) |  | (.149) |  |
| Latino | .409 | ** | -.259 |  | .330 | ** | -.126 |  | .028 |  |
|  | (.140) |  | (.144) |  | (.127) |  | (.132) |  | (.140) |  |
| Other | .188 |  | -.077 |  | .203 |  | -.153 |  | -.222 |  |
|  | (.175) |  | (.132) |  | (.159) |  | (.172) |  | (.181) |  |
| Fully online learning |  |  | -0.174 | ** | -.169 | ** | -.061 |  | .005 |  |
|  |  |  | (.056) |  | (.060) |  | (.067) |  | (.067) |  |
| Number of confidants |  |  |  |  |  |  | -.101 | * | -.090 |  |
|  |  |  |  |  |  |  | (.049) |  | (.049) |  |
| Sleep quality |  |  |  |  |  |  | .571 | *** | .612 | *** |
|  |  |  |  |  |  |  | (.046) |  | (.044) |  |
| Age | .082 |  | .018 |  | .06 |  | .015 |  | .022 |  |
|  | (.054) |  | (.045) |  | (.049) |  | (.051) |  | (.054) |  |
| Female | -.009 |  | -.029 |  | .240 | ** | .321 | ** | .180 |  |
|  | (.109) |  | (.087) |  | (.093) |  | (.099) |  | (.104) |  |
| Annual household income (logged) | -.042 |  | .034 |  | .059 |  | -.071 |  | -.035 |  |
|  | (.057) |  | (.050) |  | (.056) |  | (.053) |  | (.052) |  |
| Household size | -.039 |  | .010 |  | -.100 | * | .136 | ** | .071 |  |
|  | (.054) |  | (.048) |  | (.048) |  | (.050) |  | (.053) |  |
| Lives in metropolitan area | .245 |  | .083 |  | -.036 |  | -.144 |  | .050 |  |
|  | (.148) |  | (.114) |  | (.127) |  | (.135) |  | (.137) |  |
| Has broadband internet | .052 |  | .220 |  | .005 |  | .346 |  | .086 |  |
|  | (.183) |  | (.190) |  | (.137) |  | (.180) |  | (.190) |  |
| Note: Numbers in parentheses are standard errors. Model is just identified. | | | | | | | | | | |
| *p < .05, **p < .01, and ***p < .001 (two-tailed tests). | | | | | | | | | | |

| **Table S3.** Standardized estimates for path model with demographic variables and state-level variables entered as covariates for each path. | | | | | | | | | | |
| --- | --- | --- | --- | --- | --- | --- | --- | --- | --- | --- |
|  | Dependent Variable | | | | | | | | | |
| Independent variable | Fully online learning |  | Number of confidants |  | Sleep |  | Anxiety |  | Depression |  |
| Ethnoracial identity (vs. White) |  |  |  |  |  |  |  |  |  |  |
| Black | .588 | *** | .054 |  | .007 |  | .003 |  | -.031 |  |
|  | (.161) |  | (.137) |  | (.144) |  | (.155) |  | (.156) |  |
| Latino | .353 | * | -.288 |  | .315 | * | -.210 |  | -.017 |  |
|  | (.146) |  | (.151) |  | (.130) |  | (.144) |  | (.142) |  |
| Other | .152 |  | -.125 |  | .204 |  | -.182 |  | -.215 |  |
|  | (.178) |  | (.135) |  | (.161) |  | (.179) |  | (.199) |  |
| Fully online learning |  |  | -0.207 | *** | -.176 | ** | -.056 |  | .002 |  |
|  |  |  | (.057) |  | (.061) |  | (.069) |  | (.071) |  |
| Number of confidants |  |  |  |  |  |  | -.097 | * | -.084 |  |
|  |  |  |  |  |  |  | (.049) |  | (.049) |  |
| Sleep quality |  |  |  |  |  |  | .563 | *** | .593 | *** |
|  |  |  |  |  |  |  | (.047) |  | (.046) | *** |
| Age | .078 |  | .078 |  | .068 |  | .020 |  | .020 |  |
|  | (.054) |  | (.054) |  | (.057) |  | (.051) |  | (.055) |  |
| Female | -.004 |  | -.022 |  | .242 | ** | .328 | ** | .191 |  |
|  | (.109) |  | (.088) |  | (.093) |  | (.102) |  | (.107) |  |
| Annual household income (logged) | -.041 |  | -.041 |  | .062 |  | -.080 |  | -.032 |  |
|  | (.056) |  | (.056) |  | (.057) |  | (.055) |  | (.054) |  |
| Household size | -.048 |  | -.048 |  | -.081 |  | .150 | ** | .078 |  |
|  | (.054) |  | (.054) |  | (.048) |  | (.051) |  | (.055) |  |
| Lives in metropolitan area | .197 |  | .070 |  | -.052 |  | -.195 |  | .029 |  |
|  | (.152) |  | (.125) |  | (.129) |  | (.140) |  | (.139) |  |
| Has broadband internet | .050 |  | .231 |  | -.058 |  | .336 |  | .082 |  |
|  | (.183) |  | (.185) |  | (.166) |  | (.174) |  | (.194) |  |
| Note: Numbers in parentheses are standard errors. Model is just identified. | | | | | | | | | | |
| *p < .05, **p < .01, and ***p < .001 (two-tailed tests). | | | | | | | | | | |
